# Approaching physically active learning as a multi, inter, and transdisciplinary research field

**DOI:** 10.3389/fspor.2023.1228340

**Published:** 2023-07-28

**Authors:** Mathias Brekke Mandelid

**Affiliations:** ^1^ Department of Sports, Physical Education and Outdoor Studies, Faculty of Humanities, Sports and Educational Science, University of South-Eastern Norway, Bø, Norway; ^2^ Center for Physically Active Learning, Faculty of Education, Arts and Sports, Western Norway University of Applied Sciences, Sogndal, Norway

**Keywords:** physically active learning, multidisciplinary, interdisciplinary, transdisciplinary, movement integration

## Abstract

In broad terms, physically active learning is a phenomenon that combines health and educational disciplines to integrate physical activity and core educational goals. Despite a growing research interest within the physically active learning field, conceptual clarity on combining and synthesising research disciplines appears to be needed. This article thus explores knowledge production within the physically active learning research field. First, it outlines the origin of the research field. Secondly, the terms multi-, inter-, and transdisciplinary are applied to confront how knowledge is produced. Finally, the three approaches' theoretical and ethical implications are discussed. The article contributes to conceptual clarity within the field by proposing that physically active learning is inherently inter- and transdisciplinary because it embraces the complexity of integrating and synthesising knowledge from health and educational disciplines to address real-world problems. To conclude, awareness of physically active learning's practical and theoretical dimensions through the three approaches is central to evolving the field.

## Introduction

During the last two decades, increasing political interest has emphasised schools as a privileged context to promote physical activity (PA) because it presents an arena to reach young people from all social, cultural, and economic groups (1). For instance, the PA strategy for the World Health Organization European Region 2016–2025 (2) and the Global Action Plan on PA 2018–30 (3) highlight the need to strengthen teachers' competence and create school environments encouraging young people to be physically active. This increasing interest has provided a fundament for researchers to implement numerous initiatives that promote PA during the school day. Historically, many initiatives have sought to promote PA within the context of physical education (PE). However, as the curriculum pressures time dedicated to PE, there has been a growing recognition for providing PA opportunities across all areas of the school day (4). Such interventions have sought to increase PA opportunities through strategies such as active transportation, longer recess, or improved levels of PA. More recently, there has been an interest in exchanging sedentary activities with physical activity by adding short breaks where pupils are physically active, either with curriculum content or without (5). The reinforcement of combining PA with educational activities has also led to the integration of PA into lessons in theoretical subjects. Despite these PA initiatives, relatively few have demonstrated changes in the targeted mediators (6). Two meta-analyses have even gone as far as challenging the notion of school as the optimal arena because the results indicate that most programs are ineffective and lack sustainability (7, 8).

Concurrently, schools are complex and situated in an ever-changing educational landscape of competing interests (9). With few exceptions, an unintended consequence of contemporary education is that the attention to measurable outcomes and teaching to the test have contributed to pupils becoming ever more sedentary because there is a perception that learning occurs at the desk (10). Such educational development might be challenging for designing school-based PA initiatives that support education contexts. In this context, play-based learning and physically active play exemplify fields that integrate more holistic learning and development with the curriculum (11, 12). Another research field that has grown out of a need to overcome competing interests between academic pursuits vs. time spent on PA while addressing core educational goals is Physically Active Learning (PAL) (13). In this vein, PAL combines health and educational disciplines by allowing researchers to investigate young people's PA levels and learning.

For this article, the term discipline describes a particular body of knowledge that can be taught or learned (14). For instance, health disciplines within the PAL research field refer to sub-disciplines such as health promotion, medicine, epidemiology, and psychology. Simultaneously, the educational disciplines refer to pedagogy and learning theories. Each discipline has practices that apply the body of knowledge and methods that provide a legitimate and epistemologically coherent body of theory (15). While disciplines describes the body of knowledge, the term field refers to research practices that study the disciplines (16).

In broad terms, combining disciplines has been widely applied to synthesise knowledge from various academic and non-academic disciplines to solve society's complex and dynamic problems (17). The PAL research field is no exception; combining the two disciplines is motivated by solving a complex real-world problem involving young people's health and learning without taking away time from either. However, combining these disciplines may be challenging as researchers claim that the epistemological body of theory within the health discipline may contest the purposes of education (18). In general, combining disciplines has important implications which warrant consideration. For example, some methodologists associate opening boundaries between disciplines with artful sustainability and new ways of producing knowledge (19, 20). In contrast, others claim that it poses specific problems and advocate for maintaining and protecting the integrity of disciplinary boundaries (14).

The departure point for this article is a growing interest in the PAL field to develop new ways of structuring, addressing and reaching problems and solutions regarding young people's health and education. However, the different degrees to which the two disciplines are combined and synthesised indicate that the health discipline has led the field's progress, leaving less room for education. Based on an overview of relevant research, this article seeks to make sense of and provide conceptual clarity of knowledge production within the PAL field. The idea of multi, inter, and transdisciplinarity has become more commonplace in the broader health and educational literature because research needs to rethink approaches to real-world problems (21–24). The current article, therefore, draws on these particular terms to sort relevant PAL research into ways of approaching the field based on their combining and synthesising of disciplines. Further, the article confronts the PAL research field and the somewhat taken-for-granted combination and synthesising of disciplines by suggesting that PAL are inherently inter, and transdisciplinary. Then theoretical and ethical implications for practices within the PAL research field are discussed. By doing so, the article offers ways of approaching PAL and opens a space for more dialogue and reflection on research practices.

## Approaching physically active learning as a multidisciplinary research field

**Figure 1:**
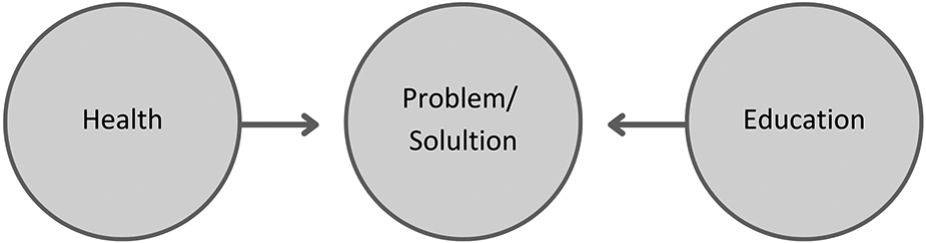
Approaching physically active learning as a multidisciplinary research field (26).

Following is a conceptualisation of knowledge production when approaching PAL as a multidisciplinary research field. Multidisciplinary refers to many, multiple or more than one existing discipline (14). In other words, multidisciplinary draws on knowledge from different disciplines but stays within its methodological boundaries to investigate a problem or a solution (25). Additionally, the language of the respective disciplines persists and remains unaltered (15). In this view, PAL is not a relevant term because it alters the language of the disciplines. Therefore, to maintain consistency and clarity throughout the rest of this article, the term classroom-based PA refers to combining health and educational disciplines within their methodological distinctiveness (see Figure 1).

As mentioned, combining health and education disciplines to integrate PA into educational goals has emerged out of the health field. Although PA has many motives and benefits, health has been the dominant rationale. Indeed, research on classroom-based PA and PAL seems to be no exception, as the research field utilises PA ubiquitously for health (5). One reason is that classroom-based PA has been pursued by health researchers who design methodological approaches for school interventions (23, 27). As a result, these methodological approaches have focused on measuring pupils' levels of PA and investigating classroom-based PA's contribution to improving, among others, physical fitness, cognitive function and academic performance (28).

This approach to classroom-based PA resonates with multidisciplinary research because researchers maintain each discipline's methodological distinctiveness (15). For instance, multidisciplinary research on classroom-based PA is often conducted through cross-sectional, acute, longitudinal or intervention trials (28). Furthermore, researchers use predefined parameters such as sedentary time or light, moderate to vigorous PA to investigate intensity levels. Accelerometers, aerobic fitness tests and shuttle run tests are examples of measurements for pupils' capacity for intense activity (29). In multidisciplinary research, objective measures enable researchers to investigate how PA can support or benefit cognitive functions. For example, two recent systematic reviews and meta-analyses show that standardised reading, spelling, or math tests are common measures of cognitive function and academic outcomes (5, 30).

The health rationale appears to have dictated research areas and knowledge production within the PAL research field, giving learning a narrow focus with little attention given to broader educational purposes. Consequently, multidisciplinary research practices in the classroom-based PA field utilise distinct methodological approaches to integrate PA to promote and enhance cognitive processes to establish an evidence base of benefits.

## Approaching physically active learning as an interdisciplinary research field

**Figure 2:**
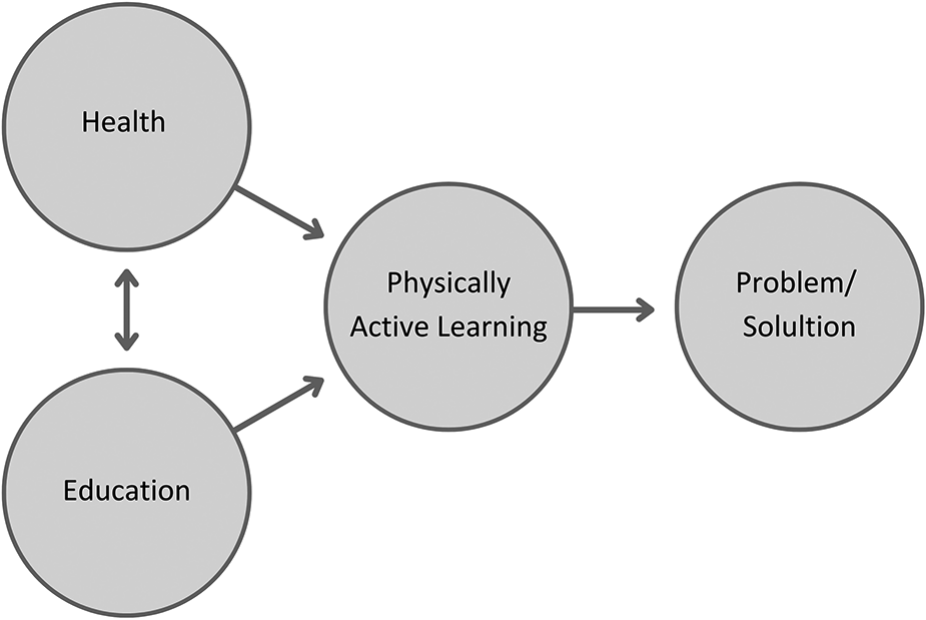
Approaching physically active learning as an interdisciplinary research field (26).

Following is a conceptualisation of knowledge production when approaching PAL as an interdisciplinary research field. The term inter means reciprocal, mutual and among (14). Interdisciplinary, thus, refers to several existing disciplines with a reciprocal relationship that links into a coherent whole (25). Furthermore, interdisciplinary research allows the creation and modification of hybrid solutions (15). Interdisciplinary research practices thus enable researchers to synthesise health and education in a reciprocal relationship, creating physically active learning, a partly new discipline with the opportunity for new practises (see Figure 2).

The term PAL implies interdisciplinarity because it consists of physical activity and learning. More precisely, it refers to being physically active *while* learning. This distinction is central to approaching PAL as an interdisciplinary research field because it does not only refer to combining the two disciplines but synthesising them. Although the health discipline has been prioritised over education, interdisciplinary research has gained interest because researchers, similar to the broader PA field, are concerned with allowing more complex opportunities (21, 22, 31–33).

This change of interest within the PAL research field has broadened alternative research methods where a more comprehensive understanding of the teachers' role, adaptation and integration of PAL into everyday pedagogical practices have become central. For instance, the definition “the integration of movement into the delivery of academic content” exemplifies that movement has become a more frequently used term that opens up ways of researching PA (34). Evident examples in the field are projects that include teachers' perspectives and experiences on the purpose of the movement in educational settings and the feasibility of integrating PAL. For example, although some researchers use the methodological distinctiveness of health disciplines to design PAL interventions, an interdisciplinary approach enables them to extend their conceptual reference or theoretical framework (15). A recent meta-synthesis (34) shows that relevant theoretical frameworks extending the field include the Socio-Ecological Model (35), the COM-B model (36) and the RE-AIM framework (37). These frameworks map and evaluate teachers' motivation, behaviours and practices using qualitative and quantitative methods such as interviews, focus groups and questionnaires (34, 38–41).

Approaching PAL as an interdisciplinary research field has also given rise to education by offering reciprocity between the two disciplines. In particular, interdisciplinarity has given rise to the theoretical reciprocity of the disciplines (14), which has oriented knowledge production toward pedagogy and didaktikk and a more layered understanding of teachers' perceptions of and experiences with PAL (10, 40). For instance, such research prioritises teachers' understanding of what movement contributes to teaching to refocus on the broader purposes of education, such as pupils' learning, social development and identity formation. Moreover, this research moves beyond health and educational disciplines as separate entities to explore them as one.

Interdisciplinarity enables researchers to synthesise the disciplines' methodologies to produce knowledge by extending its conceptual and theoretical frameworks. Moreover, it broadens the field by giving rise to investigations of the purpose of movement, the feasibility of teachers’ adaptation and the educational contexts to a larger degree. In addition, bringing diverse health and education insights into a conversation gives new opportunities to the research practice of PAL.

## Approaching physically active learning as a transdisciplinary research field

Following is a conceptualisation of knowledge production when approaching PAL as a transdisciplinary research field. The term' trans' means beyond, through and change (14). Transdisciplinary refers to going through or beyond each discipline. Furthermore, a transdisciplinary approach differs from multi- and interdisciplinary in going beyond the boundaries between disciplines for social purposes, resulting in problem-solving in the real-life world to evolve and adapt practices continuously (26).

**Figure 3:**
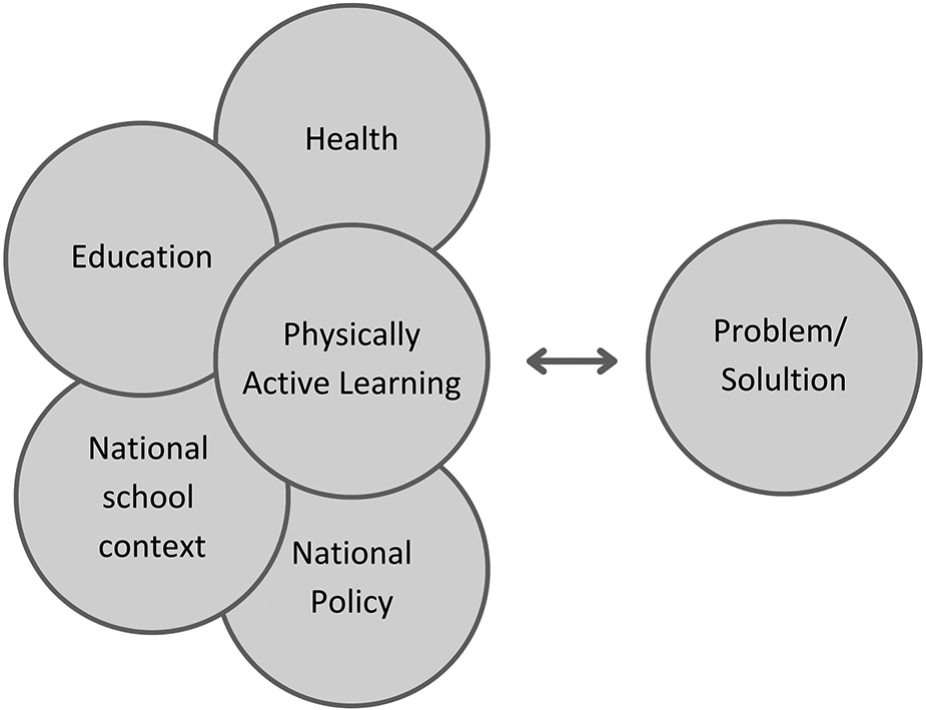
Approaching physically active learning as a transdisciplinary research field (26).

Although only a small body within the PAL research field deals with transdisciplinarity, there is an ever-increasing interest in moving beyond research-led approaches to evolve PAL. This part of the field is drawing on a rationale for broadening research approaches that place PAL in a reciprocal relationship between practice and theory to justify holistic perspectives of PA and active learning methods in education (10, 40, 42). Approaching PAL as a transdisciplinary research field thus enables health and educational disciplines to be addressed in a reciprocal relationship between theory and practice (see Figure 3). Unlike other approaches to research, theory and practice in transdisciplinary research continuously address and inform each other (15).

The co-production between academics and non-academics that unify theory and practice is thus common to knowledge production when approaching PAL as a transdisciplinary field. Although researchers traditionally have had an analytical distance from research projects, transdisciplinary research embraces academic and non-academics' mutual participation in co-producing knowledge (19, 23). Examples in the PAL research field include research on system design (38, 39, 43, 44) and empirical queries about planning, designing and developing PAL in teaching (45). On a system design level, the co-productive stakeholder perspectives aim to understand PAL's design, adoption and integration. In addition, it informs an understanding of PAL beyond classroom settings and raises the importance of school- and national-level contextual factors (38).

Further, on empirical queries about planning, designing and developing PAL in teaching, one project included teachers as collaborators in designing PAL activities (43, 45). Engaging teachers in this way also means being open for health and educational disciplines to be broadened and synthesised into new languages that can express relevant concepts (15). For instance, one study used a new term, “enactive movement integration”, to express the phenomenon of combining health and educational disciplines (45).

Knowledge production within transdisciplinary research means solutions contribute to the disciplines it drew upon to evolve them continuously. Like the broader field of PA, one reason for the growing transdisciplinary interest in PAL is that it is considered a domain in research, policy and practice which is rooted in addressing real-life problems (14, 39, 44). Although approaching PAL as a transdisciplinary research field is grounded in empiricism, knowledge production also needs sound theoretical frameworks to identify components and structures (23). Transdisciplinary integrates natural, social and health science in the humanities context and, in doing so, enables frameworks that articulate problems and solutions that often transcend traditional boundaries (14). Participatory action research and design thinking are frameworks utilised in the aforementioned examples of transdisciplinary research that exemplify how new language can be co-produced (46, 47).

Approaching PAL as a transdisciplinary research field enables researchers to go beyond and between health and education. It invites non-academics perspectives to structure, address, and reach problems and holistic solutions about pupils' health and learning. Such a process implies that producing and accumulating knowledge cannot be reduced to techniques and methods that defend boundaries. Instead, it breaks down the existing compositions into their elements and recombines them into new forms. That is, rethinking holistic approaches to human movement and educational purposes within the dynamics of whole systems.

## Embracing the complex knowledge production of physically active learning

The terms multi, inter, and transdisciplinarity have offered three approaches presenting the methodological breadth and depth of knowledge production in the PAL research field. Against the three approaches, previous multidisciplinary research has prioritised health and left less room for education. This limitation to consider the educational disciplines originates from a health rationale that has given methodological prioritisation to a positivistic paradigm (48). Such a methodological prioritisation seems to stem from designing programs focusing on large-scale trials and generalisability by ensuring the effectiveness of the intervention. These ways of producing knowledge are criticised for being simplistic because they are tightly constructed, single-component programs with limited capacity to address the relationship between science and society in its design and evaluation (49). These debates have led to a resurgence in embracing the complexity of knowledge production within social and cultural contexts to enable opportunities which span disciplinary boundaries (19).

Moreover, the positivistic paradigm encapsulates the body and mind as dualistic entities enabling objective measures of PA. In line with defining PA as “any bodily movement produced by skeletal muscles that result in energy expenditure” (22, 50), a key challenge with multidisciplinary approaches is that PA might become instrumental because it is device-measured and does not include the subjectives’ broader perspectives on movement (21). The need to include context, that is, relevant actors, demographic and social and cultural factors, has, led researchers to offer a new, holistic PA definition: “people moving, acting and performing within culturally specific spaces and contexts, and influenced by a unique array of interests, emotions, ideas, instructions and relationship” (22). Even though no known PAL research has used this PA definition, similar holistic approaches have become more common in exploring experiences and perceptions of PA and movement related to learning (32, 34).

By confronting knowledge production in the PAL research field, this article proposes that PAL is inherently inter- and transdisciplinary because it offers a basis to embrace the complexity between health and education. Theoretically, the proposal suggests that PAL differs from classroom-based PA in that it does not only combine health and educational disciplines but integrates and synthesises them. In line with the new, holistic PA definition, approaching PAL as an interdisciplinary research field implies a constructivist paradigm (22, 51). This paradigm suggests that the body and mind are one system and must be interpreted and discovered in underlying meaning and activities. Such a view reprioritises participants' experiences and perceptions as important contributors to evolving PAL. From a transdisciplinary approach, PAL is constantly negotiated, debated and interpreted in light of its real-world usefulness. This pragmatic paradigm embraces the complexity of knowledge production within the field by changing its problems, methodology and collaboration in and between policy, practice and research (52). Consequently, proposing that PAL is inter- and transdisciplinary to overcome the limitations of multidisciplinary research implies that knowledge production in health and education is equally valued. Additionally, it means that a broader range of actors are engaged and included based on their knowledge and experience in diverse stages of research. Such holistic epistemologies have the potential to unify health and education in PAL into queries about pupils' learning, development and growth.

## Discussion

Proposing that PAL is inter- and transdisciplinary might disrupt a multidisciplinary research practice by emphasising its holistic nature. Theoretically, this disruption refers to the distinction between objectivity and subjectivity regarding the disciplinary structures that enable researchers to examine and describe PAL.

While some researchers argue that breaking down boundaries between health and educational disciplines has the potential to deal with the complexity of knowledge production, others claim that boundaries are essential to frame and measure a problem (53). Boundaries and definitions serve research functions as epistemological markers by providing a specific language (24). For instance, approaching PAL as a multidisciplinary field through the optics of a positivistic paradigm determines PAL based on its measurable study object. By utilising this approach, PAL is conceptualised into parameters such as intensity, frequency and duration and determined academic content that researchers decide.

Therefore, proposing that PAL is inherently inter- and transdisciplinary moves the field beyond a reality where PAL is only determined by its study object and legitimated by an epistemologically coherent body of theory (15, 19). Rather than defining its boundaries, an inter- and transdisciplinary approach enables a subjective worldview where PAL is not a set of rigid rules or hypotheses that observations can confirm. Instead, PAL is the reality of those who partake in it by allowing normative desires and questions of what it can be. In this vein, an inter- and transdisciplinary approach is more open-ended, allowing education as a context and specific practices to shape and construct PAL (20, 52). Such a proposal also indicates that the development of PAL lies in the nexus between theory and practice.

The increasing engagement of relevant non-academic's roles in co-producing knowledge about PAL has oriented the field toward a real-world understanding of what PAL is and can do. Some researchers have labelled the increasing participation of non-academics in the research processes mode-1 and mode-2 science (54). Mode-1 and mode-2 science are helpful by providing terms to distinguish between research *on* and *with* participants (24). Because a large body of PAL research is research-led and governed by academic interests in designing and conducting research, mode-1 science is the most common way of producing knowledge on participants (55). Multi- and interdisciplinary approaches belong to mode-1 science (54).

To overcome the existing limitations of multidisciplinary interventions in school, some researchers argue that a collective focus on health parameters may not be appropriate for designing and conducting research. Researchers argue that there is a need to move away from tightly constructed, single-component interventions and that progression should include the school context and its participants (22, 24, 49). Mode-2 science moves beyond these researcher-led approaches by enabling academics and non-academics to co-produce knowledge that illuminates real-world problems (23, 54). In this way, knowledge production differs from the interventions mentioned above in that knowledge is produced in the context of its application. Therefore, a transdisciplinary approach to PAL might benefit flexible research that enables researchers to apply a range of theoretical perspectives and methodologies to describe the practices in which PAL is enacted. For example, PAL research accounting for the relationship between social and cultural contexts and education's broad scope indicates a more layered insight into teacher adaptation of PAL that derives from the enactment in everyday practice and school context (45). In this way, a transdisciplinary approach might be beneficial to move beyond a collective focus on health parameters and turn attention to relevant actors and education.

Although mode-2 science implies a less hierarchical relationship by inviting academics and non-academics to co-produce knowledge, it raises democratic considerations about producing holistically and socially robust knowledge about PAL (19, 20). On the one hand, involving academics and non-academics in discussions about the conditions and opportunities regarding PAL can lead to problem-solving in school contexts. On the other hand, it can lead to epistemic development that contests the traditional disciplinary boundaries by creating new languages. For instance, when describing PAL, teachers use an educational language that leaves out health disciplines (10, 56). These findings suggest that knowledge production depends on diverse constellations of expertise that can bring together health and educational disciplines. A sole focus on transdisciplinary approaches might challenge multidisciplinary research because knowledge production does not belong to a coherent body of theory that stays within its boundaries. Therefore, approaching PAL as a transdisciplinary research field implies a reflexive and transformative exploration of practices that are not clearly defined (16).

In this way, a key point for evolving PAL is awareness regarding the distinction between objectivity and subjectivity in research. Rather than an either-or perspective, moving beyond the current limitations of PAL intervention might be coupled with its practical and theoretical dimensions. Even though multidisciplinary approaches primarily prioritise objective measures and parameters that derive from the theoretical dimensions of a health and educational discipline, there is also a need to be reflexive regarding what PAL looks like in a complex and dynamic landscape such as school contexts. Equally, although inter- and transdisciplinary approaches have the potential to be context specific and produce new opportunities for PAL, it is central to account for its theoretical foundation. In line with mode-2 science, reflexivity involves awareness of the phenomenon, in this case PAL, so that it does not lose its form or become a simplification of reality (16, 20). On this note, transdisciplinary research remains silent on becoming “theories of everything” at the expense of the coherence and consistency of the disciplinary methods and knowledge production (14). Therefore, reflexivity means the willingness to embrace the complexity by letting the participants and context shape the research interests. Embracing the complexity also means that PAL researchers must be transformative regarding combining and synthesising disciplines and the theories and methodologies used to discuss what PAL is and can do.

## Conclusion

This conceptual article has offered three approaches to the PAL research field. By confronting how health and educational disciplines are combined and synthesised to produce knowledge, the article's author proposes that PAL is inherently inter- and transdisciplinary because it offers a foundation for researchers to embrace the complexity of integrating and synthesising knowledge from health and educational disciplines. Yet, this does not suggest that the methodological structures in health and educational disciplines should be ignored or disappear. Rather than offering one definite way of combining and synthesising the field, proposing that PAL is inter- and transdisciplinary is meant to contribute to increased awareness about opportunities for researchers to create theoretical structures, questions and knowledge that can deal holistically with pupils' growth beyond the guarded boundaries of the existing disciplines. Reflection on PAL's practical and theoretical dimensions and what it means to approach it as a multi, inter, and transdisciplinary field is central to evolving the research practice in the field.
